# Dinner or Lunch

**Published:** 1875-09

**Authors:** 


### DINNER OR LUNCH?

It is a custom which prevails among business men in large cities, to eat at night. The idea obtains with many that the principal meal should be taken in the middle of the day, and that the evening repast, as well as the breakfast, should be light. This is the practice with farmers and people in the country, and in country villages. With them it is practicable. The farmer takes his "nooning;" and a quiet hour or two after eating a leisurely meal is wonderfully conducive to a healthy digestion. The country merchant is not likely to be so driven with business that he has not the time to get his "regular dinner" and his after dinner smoke, if he is fool enough to smoke at all.

But the business man in a great city is under whip and spur from 9 o'clock in the morning until 3 or 4, or 5 in the afternoon. There is no place in the world where men work harder than they do in New York. The labor of the day is mostly compressed into the space of six hours, and the busiest part is that which old-fashioned people were won't to devote to dinner time.

Though it might be very agreeable, it would be quite impracticable for the New York merchant or banker, or broker or editor to leave his business and go to Brooklyn or Jersey or up-town to take his mid-day dinner at home with his family. And so, necessity compelled men to adopt the custom of dining at 6 o'clock and lunching any time from 12 till 2. This is, we believe, much the better way for the busy New Yorker. It would be for him a very injurious practice to eat a full dinner at 1 o'clock, and then in the next 2 or 3 hours perform the hardest and most exciting part of the labor of the day. His whole energies, physical and mental, are directed to his business. At such a time the process of digestion can be but imperfectly performed—if indeed it is not nearly suspended. But the food in the stomach if not being digested is undergoing decomposition, with the evolution of gas and the formation of acid. Hence flatulence, pain, heartburn, dyspepsia.

While we regard a regular dinner in the midst of the business hours of the day as injurious, we would not advise any man to go without food from morning to night. The proper course is to eat a good breakfast and then take a light, easily digested lunch at 12 or 1 o'clock. A "hasty plate of soup," a dish of bread and milk, or of oatmeal mush and milk, or any of the palatable and wholesome farinaceous preparations will "stay the stomach" without overloading it.

And here we must congratulate the legion of our fellow lunch-takers on the good work that has been done in this city during the past 3 years in the establishment of those eating-rooms known as "dairies." We have had personal and happy experiences in some of them. We have found milk as fresh and sweet and rich as we ever drank in the days when "we boys" used to go after the cows at night. We have found cream, genuine cream, giving its color in the cups of coffee and tea, with that luscious flavor which nothing else can afford to these refreshing beverages. To those accustomed to them, tea or coffee with the light lunch is gently stimulating, and serves to support the nervous system against the exhaustive wear and tear of city business life.

We would, then, recommend regularity in the times of eating; the breakfast to be substantial, the mid-day lunch to be considered as important in its place as any other meal and to consist of light and digestible food, and the dinner to be a reasonable repast, and to be eaten—as it must be by city men—after the labor of the day is ended, say at 6 o'clock, leaving several hours for the work of digestion before a decent bed-time.

